# Supplementary material for: Hexanary blends: a strategy towards thermally stable organic photovoltaics
Source: Nat Commun. 2023 Aug 1;14:4608. doi: 10.1038/s41467-023-39830-6 (PMC10393981; doi:10.1038/s41467-023-39830-6)
Supplement: Supplementary file 1 — Supplementary Information [file 41467_2023_39830_MOESM1_ESM.pdf]

Supplementary Information

# Hexanary Blends: A Strategy Towards Thermally Stable Organic Photovoltaics

Sri Harish Kumar Paleti[1]*, Sandra Hultmark[2], Jianhua Han[1], Yuanfan Wen[1], Han Xu[1], Si Chen[1], Emmy Järsvall[2], Ishita Jalan[3], Diego Rosas Villalva[1], Anirudh Sharma[1], Jafar. I. Khan[1], Ellen Moons[4], Ruipeng Li[5], Liyang Yu[6], Julien Gorenflot[1], Frédéric Laquai[1], Christian Müller[2]*, Derya Baran[1]*

[1]Division of Physical Sciences and Engineering, KAUST Solar Center (KSC),
King Abdullah University of Science and Technology (KAUST),
Thuwal 23955-6900, Saudi Arabia
e-mail: paleti@chalmers.se, derya.baran@kaust.edu.sa

[2]Department of Chemistry and Chemical Engineering,
Chalmers University of Technology, Göteborg, 41296, Sweden
e-mail: christian.muller@chalmers.se

[3]Department of Engineering and Chemical Sciences,
Karlstad University, Karlstad 65188, Sweden

[4]Department of Engineering and Physics,
Karlstad University, Karlstad 65188, Sweden

[5]National Synchrotron Light Source II,
Brookhaven National Lab, Suffolk, Upton, NY 11973, USA

[6]School of Chemical Engineering,
College of Chemistry and State Key Laboratory of Polymer Materials Engineering,
Sichuan University, Chengdu 610065, P. R. China

**List of Supplementary Figures**

Fig. 1 Chemical structures 4

Fig. 2 DSC thermograms 6

Fig. 3 Fast Scanning calorimetry (FSC) thermograms 7

Fig. 4 AFM-IR images of H1 active layer blend film 9

Fig. 5 J-V curves: H1, H5, H5*, Q1 and B1 10

Fig. 6 Composition-efficiency diagram 10

Fig. 7 AFM micrographs of H5 active layer based films 12

Fig. 8 Thermal aging studies of Y-series based OSC 13

Fig. 9 J-V curves: ITIC based OSC 14

Fig. 10 Absorption spectra 14

Fig. 11 Thermal aging studies of ITIC based OSC 15

Fig. 12 Charge field analysis in fresh and aged devices 16

Fig. 13 JV curves of OSC in dark 17

Fig. 14 GIWAXS diffractograms 17

Fig. 15 J-V curves of devices with varied active layer thickness 20

Fig. 16 Thermal aging studies with varied active layer thickness 20

**List of Supplementary Tables**

Table 1 Molecular energetic values 5

Table 2 Melting enthalpies and melting temperatures 6

Table 3 Full width half maximum analysis 8

Table 4 Photovoltaic device parameters: H1 based combinations 11

Table 5 Photovoltaic device parameters: ITIC based OSC 13

Table 6 Thickness of the GIWAXS films 18

Table 7 Thickness of the active layer films 19

Table 8 Photovoltaic device parameters: fresh and aged H1, B2 devices 19

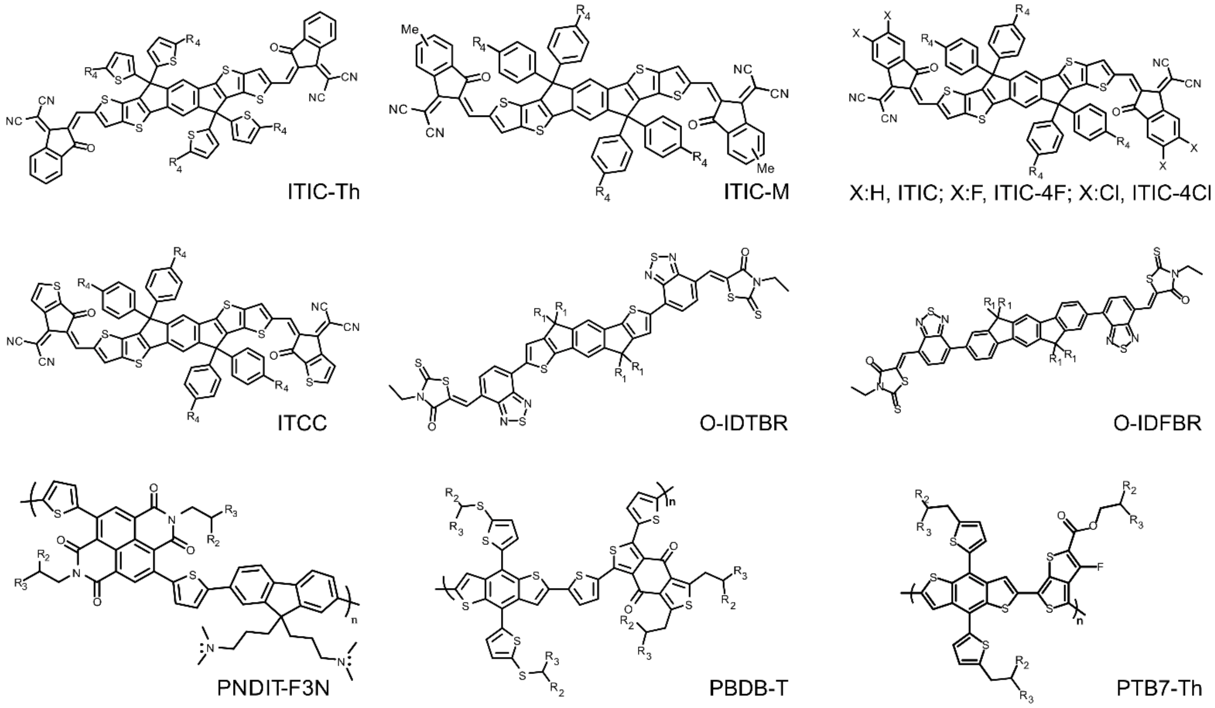


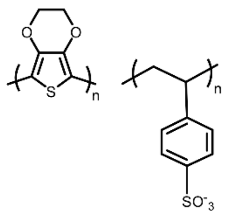

PEDOT:PSS

| Material | $R_1$ | $R_2$ | $R_3$ | $R_4$ |
|---|---|---|---|---|
| ITIC-Th | -- | -- | -- | $C_6H_{13}$ |
| ITIC-M | -- | -- | -- | $C_6H_{13}$ |
| ITIC-4H/F/Cl | -- | -- | -- | $C_6H_{13}$ |
| ITCC | -- | -- | -- | $C_6H_{13}$ |
| O-IDTBR | $C_8H_{17}$ | -- | -- | -- |
| O-IDFBR | $C_8H_{17}$ | -- | -- | -- |
| PNDIT-F3N | -- | $C_2H_5$ | $C_4H_9$ | -- |
| PBDBT (PCE12) | -- | $C_2H_5$ | $C_4H_9$ | -- |
| PTB7-Th (PCE10) | -- | $C_2H_5$ | $C_4H_9$ | -- |

Supplementary Fig. 1. **Chemical structures** of different materials mentioned in the main text.

Supplementary Table 1. **Molecular energetic values of neat materials**. The Highest occupied molecular orbitals (HOMO) values are measured by conducting photo-emission spectroscopy in air (PESA). The lowest occupied molecular orbitals (LUMO) was calculated by subtracting optical bandgap from HOMO. The absorption edge of the absorption spectra is considered as optical bandgap. The energy levels of some of the acceptor materials measured by ultraviolet photoemission spectroscopy (UPS) and low-energy inverse photoelectron (LE-IPES) spectroscopy can be found in [1] .

| Material name | HOMO (eV) | Optical bandgap (eV) | LUMO (eV) |
|---|---|---|---|
| PM6 | 5.15 | 2.06 | 3.09 |
| Y1 | 5.64 | 1.31 | 4.33 |
| Y6 | 5.78 | 1.28 | 4.50 |
| Y7 | 5.80 | 1.31 | 4.49 |
| Y11 | 5.69 | 1.28 | 4.41 |
| Y16 | 5.68 | 1.30 | 4.38 |
| Y18 | 5.70 | 1.26 | 4.44 |
| N3 | 5.79 | 1.35 | 4.44 |
| BTP-eC9 | 5.78 | 1.31 | 4.47 |
| ITCC | 5.87 | 1.70 | 4.17 |
| ITIC-M | 5.88 | 1.62 | 4.26 |
| ITIC-Th | 5.90 | 1.60 | 4.3 |
| ITIC-4F | 5.78 | 1.64 | 4.14 |
| ITIC-4Cl | 5.44 | 1.55 | 3.89 |

Supplementary Table 2. **Melting enthalpies and melting temperatures** of the different acceptor materials calculated from differential scanning calorimetry (DSC) first heating thermograms

| Acceptor materials | Enthalpy ($Jg^{-1}$) | $T_m$ (℃) |
|---|---|---|
| Y1 | 37 | 319 |
| Y6 | 29 | 297 |
| Y11 | 35 | 313 |
| Y16 | 30 | 285 |
| Y18 | 42 | 321 |
| Y6:Y11:Y16 | 14 | 282 |
| Y1:Y6:Y11:Y16:Y18 | 10 | 256 |

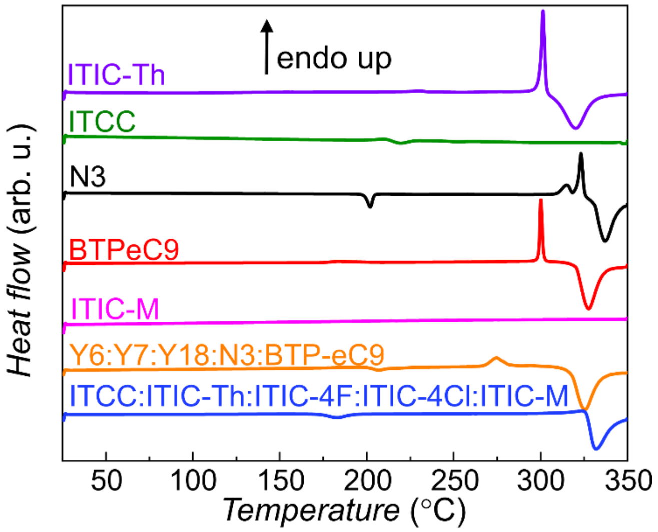


Supplementary Fig. 2. **DSC first heating thermograms** of ITIC-M, ITIC-Th, ITCC, N3, BTP-eC9, Y6:Y7:Y18:N3:BTP-eC9 and ITIC-4F:ITIC-4Cl:ITIC-Th:ITIC-M:ITCC. The DSC thermogram of ITIC-4F and ITIC-4Cl can be found in[2]. Source data are provided as a Source Data file.

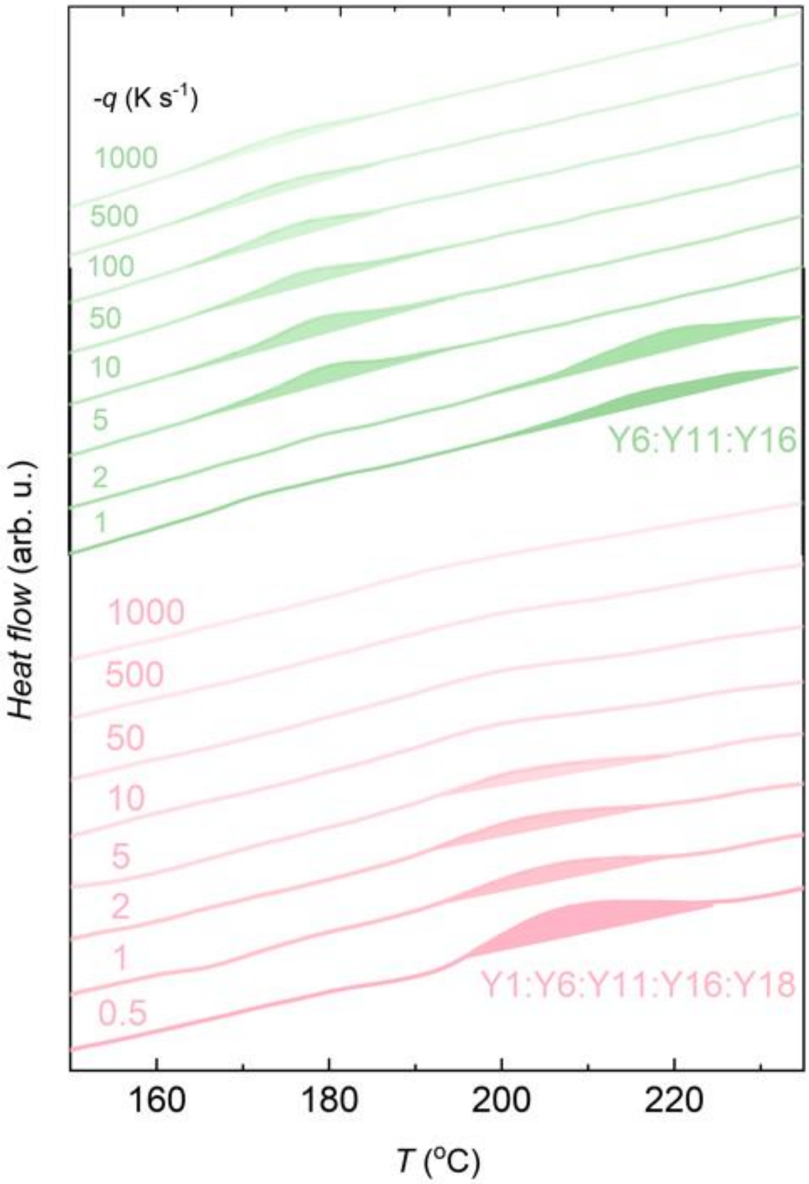


Supplementary Fig. 3. **Fast scanning calorimetry (FSC) heating thermograms** of Y6:Y11:Y16 (green trance) and Y1:Y6:Y11:Y16 :Y18 (light pink) with the enthalpy overshoot indicated by shaded areas. Source data are provided as a Source Data file.

Supplementary Table 3. **Full width half maximum analysis.** Analysis of GIWAXS patterns of as-prepared (spin-coated from chloroform and annealed for 1 min at 120 °C) and aged blend films (annealed at 130 °C for 120 h) of the binary blend B1, the quaternary blend Q1 and the hexanary blend H1: peak position in reciprocal and real space, q and d, full-width at half maximum FWHM and coherence length CL of ordered domains, calculated using the Scherrer equation.

| | | as prepared | | | aged at 130 °C for 120 h | | |
|---|---|---|---|---|---|---|---|
| | | B1 | Q1 | H1 | B1 | Q1 | H1 |
| PM6 | $q$ (nm$^{-1}$) | 2.97 | 3.04 | 3.07 | 2.97 | 3.02 | 3.06 |
| | $d$ (nm) | 2.12 | 2.07 | 2.05 | 2.12 | 2.08 | 2.05 |
| | FWHM (nm$^{-1}$) | 0.69 | 0.61 | 0.8 | 0.54 | 0.47 | 0.48 |
| | CL (nm) | 12.2 | 13.8 | 10.6 | 15.6 | 17.8 | 17.4 |
| Acceptors | $q$ (nm$^{-1}$) | 3.87 | 3.87 | 4.2 | 3.86 | 4.4 | 4.37 |
| | $d$ (nm) | 1.62 | 1.62 | 1.5 | 1.63 | 1.43 | 1.44 |
| | FWHM (nm$^{-1}$) | 0.94 | 1 | 0.99 | 0.87 | 0.31 | 0.47 |
| | CL (nm) | 9 | 8.5 | 8.5 | 9.7 | 26.6 | 18 |

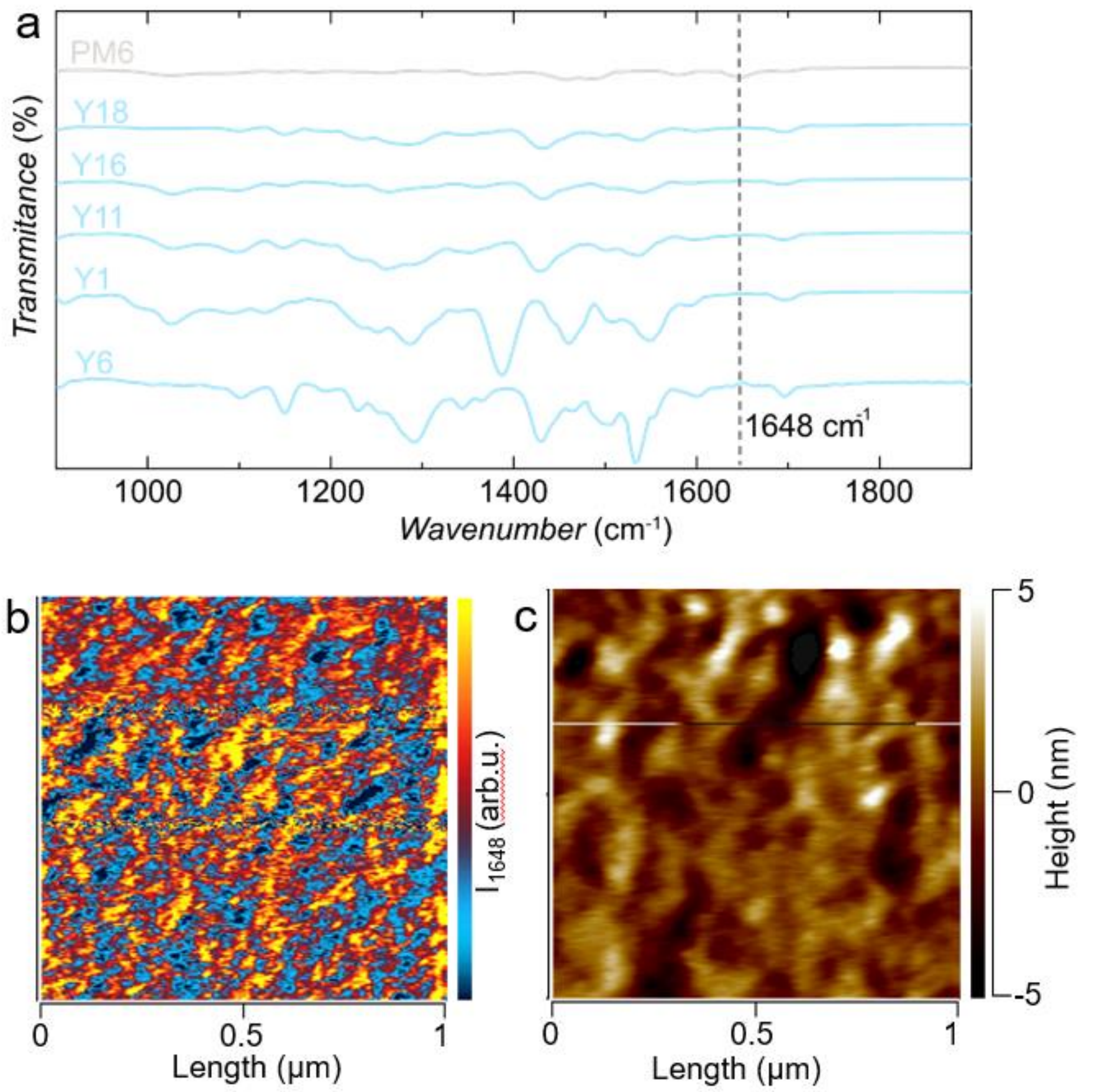


Supplementary Fig. 4. **AFM-IR images of H1 active layer blend film.** **a** AFM-IR spectra recorded for neat films of PM6, Y1, Y6, Y11, Y16 and Y18. **b** AFM image with peak-to-valley height difference and **c** AFM-IR height image of PM6:Y1:Y6:Y11:Y16:Y18 recorded at 1648 $cm^{-1}$. Source data are provided as a Source Data file.

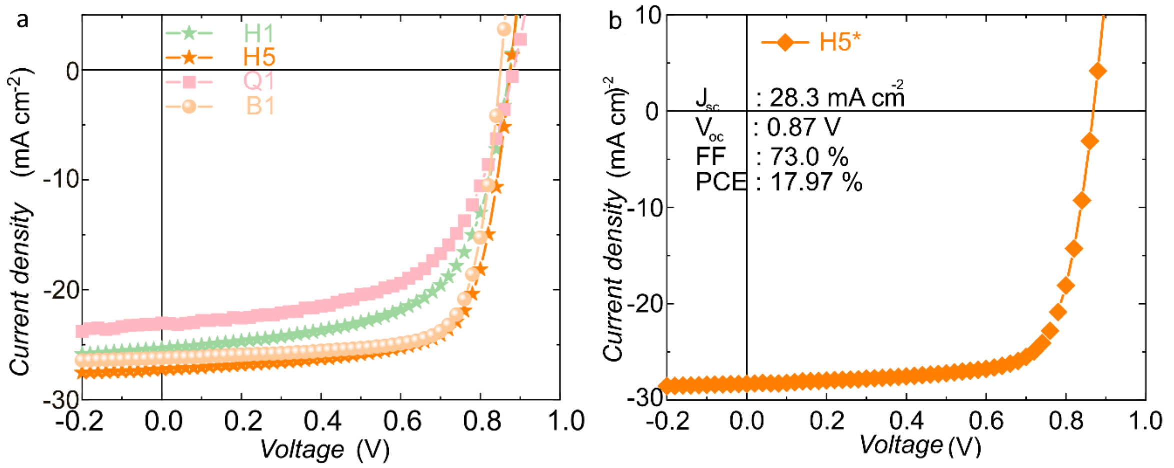


Supplementary Fig. 5. **J-V curves: H1, H5, H5*, Q1 and B1 a** The J-V curve of H1, H5, Q1 and B1 devices fabricated by spin-coating inside a glove box, and **b** the best H5* device fabricated by doctor blading in air. The J-V parameters can be found in the inset. Source data are provided as a Source Data file.

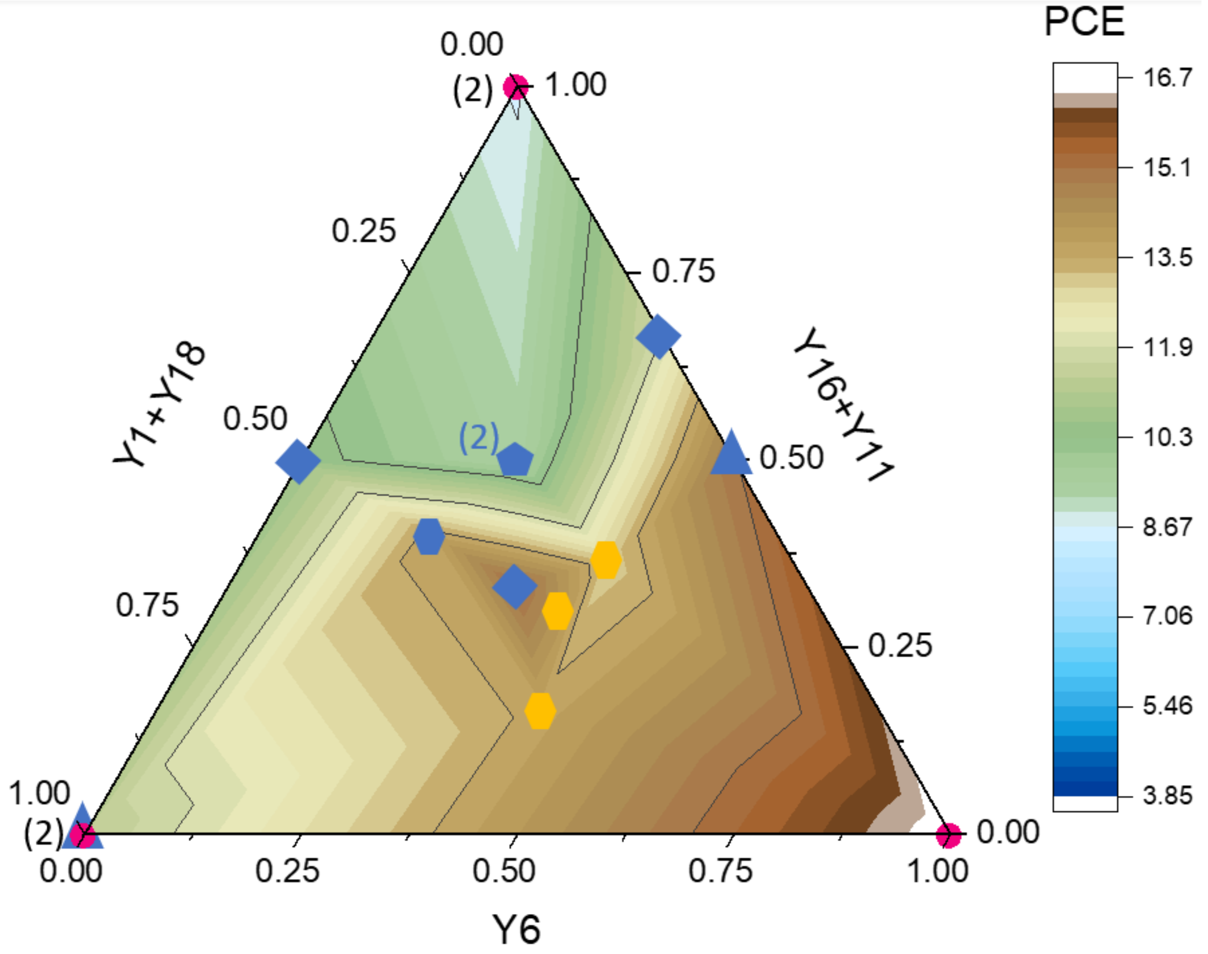


Supplementary Fig. 6. **Composition-device efficiency diagram** of binary (circles), ternary (triangles), quaternary (rhombuses), pentanary (pentagons) and hexanary (hexagons) devices based on PM6 and different combination of the acceptors Y6, Y1+Y18 and Y11+Y16. In brackets: # of compositions. The respective device parameters are given in Supplementary Table 4. Source data are provided as a Source Data file.

Supplementary Table 4. **Photovoltaic device parameters:** H1 based combinations. Mean and standard deviation of photovoltaic device parameters for 6 devices on the same substrate. B1-B5, T1-T2, Q1-Q3, P1-P2, H1-H4. The donor: acceptor ratios are weight ratios.

| Device name | Donor: acceptor ratios | $J_{sc}$ (mA $cm^{-2}$) | $V_{oc}$ (mV) | FF (%) | PCE (%) |
|---|---|---|---|---|---|
| B1 | PM6:Y6 | 26.2 ± 0.5 | 850 ± 20 | 74.9 ± 2 | 16.7 ± 0.5 |
| B2 | PM6:Y1 | 12.6 ± 0.4 | 900 ± 7 | 51.5 ± 0.8 | 5.9 ± 0.2 |
| B3 | PM6:Y11 | 24.9 ± 0.5 | 860 ± 2 | 62.0 ± 0.5 | 13.3 ± 0.3 |
| B4 | PM6:Y16 | 8.6 ± 0.1 | 980 ± 4 | 46.7 ± 0.5 | 3.9 ± 0.1 |
| B5 | PM6:Y18 | 25.7 ± 0.1 | 880 ± 5 | 68.1 ± 2 | 15.4 ± 0.2 |
| T1 | PM6:Y1:Y18 (1:0.6:0.6) | 22.0 ± 0.4 | 899 ± 2 | 64.01 ± 0.8 | 12.7 ± 0.2 |
| T2 | PM6:Y6:Y11 (1:0.6:0.6) | 26.1 ± 0.4 | 836 ± 2 | 69.07 ± 1 | 15.1 ± 0.3 |
| Q1 | PM6:Y6:Y11:Y16 (1:0.4:0.4:0.4) | 23.0 ± 0.6 | 880 ± 10 | 58.7 ± 3 | 11.9 ± 0.9 |
| Q2 | PM6:Y6:Y11:Y18 (1:0.4:0.4:0.4) | 26.3 ± 0.5 | 857 ± 7 | 67.7 ± 1.5 | 15.3 ± 0.5 |
| Q3 | PM6:Y1:Y16:Y18 (1:0.4:0.4:0.4) | 19.6 ± 0.3 | 920 ± 4 | 58.2 ± 1.4 | 10.5 ± 0.3 |
| P1 | PM6:Y1:Y6:Y11:Y16 (1:0.3:0.3:0.3:0.3) | 22.0 ± 0.1 | 902 ± 2 | 60.3 ± 0.5 | 12.0 ± 0.1 |
| P2 | PM6:Y6:Y11:Y16:Y18 (1:0.3:0.3:0.3:0.3) | 22.9 ± 0.5 | 817 ± 31 | 35.8 ± 0.9 | 6.9 ± 2.2 |
| H1 | PM6:Y1:Y6:Y11:Y16:Y18 (1:0.24:0.24:0.24:0.24:0.24) | 25.4 ± 0.3 | 880 ± 5 | 62.0 ± 1 | 13.7 ± 0.4 |
| H2 | PM6:Y1:Y6:Y11:Y16:Y18 (1:0.1:0.4:0.2:0.1:0.2) | 24.7 ± 0.3 | 871 ± 4 | 66.1 ± 2.4 | 14.2 ± 0.6 |
| H3 | PM6: Y1:Y6:Y11:Y16:Y18 (1:0.2:0.8:0.6:0.1:0.2) | 22.6 ± 0.3 | 853 ± 9 | 67.5 ± 1.7 | 13.0 ± 0.6 |
| H4 | PM6: Y1:Y6:Y11:Y16:Y18 (1:0.1:0.8:0.15:0.15:0.6) | 23.6 ± 0.9 | 855 ± 5 | 67.5 ± 2.7 | 13.6 ± 1 |

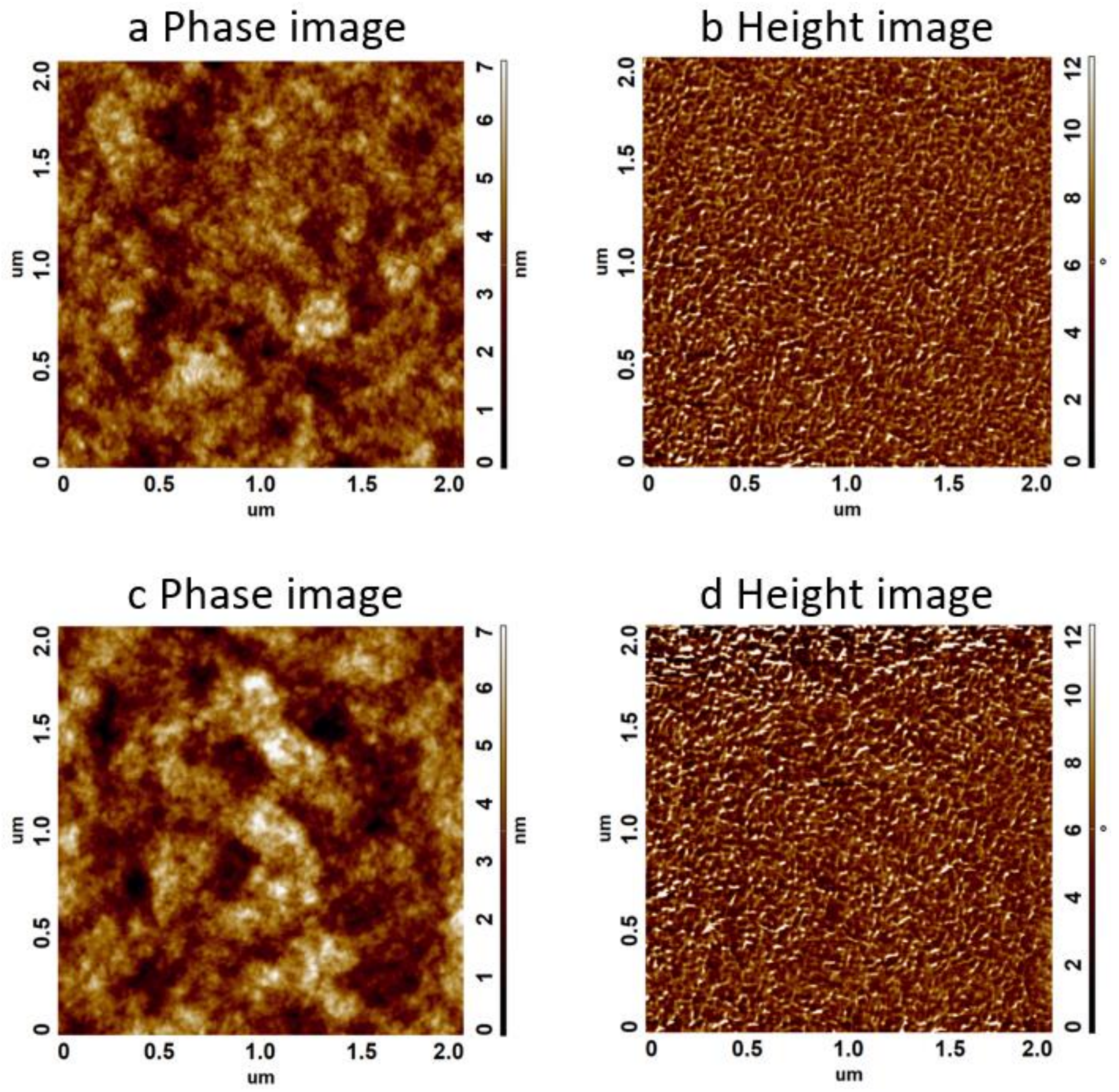


Supplementary Fig. 7. **AFM micrographs of H5 active layer based films**. Phase and height micrograph measured by atomic force microscopy of the doctor-bladed **a-b** and spin-coated **c-d** films of H5 active layer. Details are provided as a Source Data file.

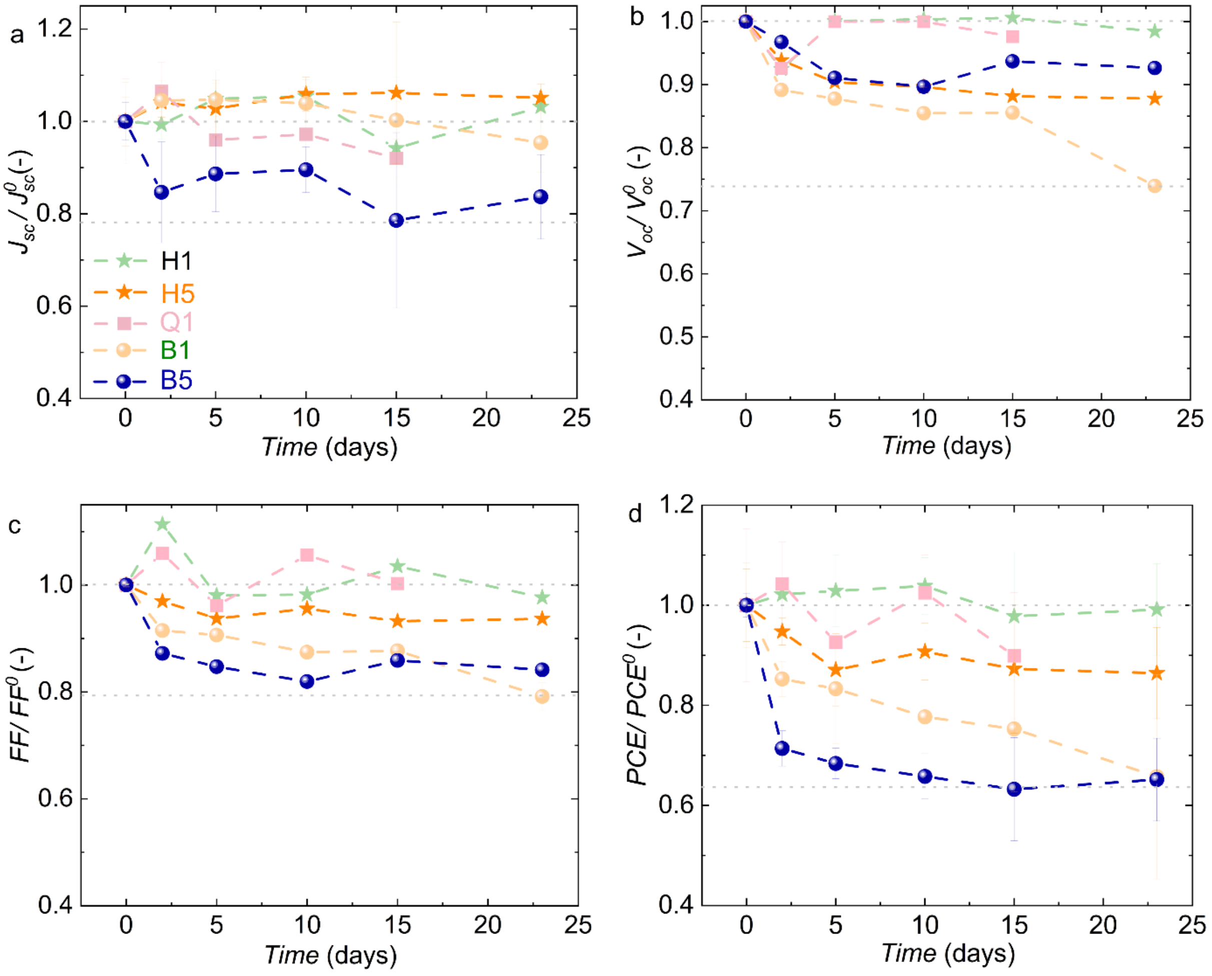


Supplementary Fig. 8. **Thermal aging studies of Y-series based OSC. a** $J_{sc}$, **b** $V_{oc}$, **c** FF and **d** PCE of H1, H5, Q1, B1 and B5 devices recorded after annealing up to 23 days at 130 °C; data points are the mean and standard deviation of measurements done for up to 6 pixels on the same substrate. Source data are provided as a Source Data file.

Supplementary Table 5. **Photovoltaic device parameters: ITIC based OSC.** Mean and standard deviation of photovoltaic device parameters for 6 devices on the same substrate. H6: PM6:ITIC-Th:ITIC-4F:ITIC-4Cl:ITIC-M:ITCC (1:0.24:0.24:0.24:0.24: 0.24); PM6:ITIC-Th (1:1); PM6:ITIC-4F (1:1); and PM6:ITIC-4Cl (1:1). The donor:acceptor ratios are weight ratios.

| Device name | $J_{sc}$ (mA cm$^{-2}$) | $V_{oc}$ (mV) | FF (%) | PCE (%) |
|---|---|---|---|---|
| Hexanary-6 (H6) | 16.7 ± 0.3 | 950 ± 8 | 57.2 ± 1 | 9.1 ± 0.4 |
| PM6:ITIC-Th (B6) | 17.8 ± 0.6 | 1001 ± 2 | 57.8 ± 1 | 10.4 ± 0.5 |
| PM6:ITIC-4F (B7) | 19.4 ± 0.3 | 910 ± 3 | 68.7 ± 1 | 12.1 ± 0.1 |
| PM6:ITIC-4Cl (B8) | 22.9 ± 0.9 | 860 ± 6 | 69.7 ± 1 | 13.7 ± 0.4 |

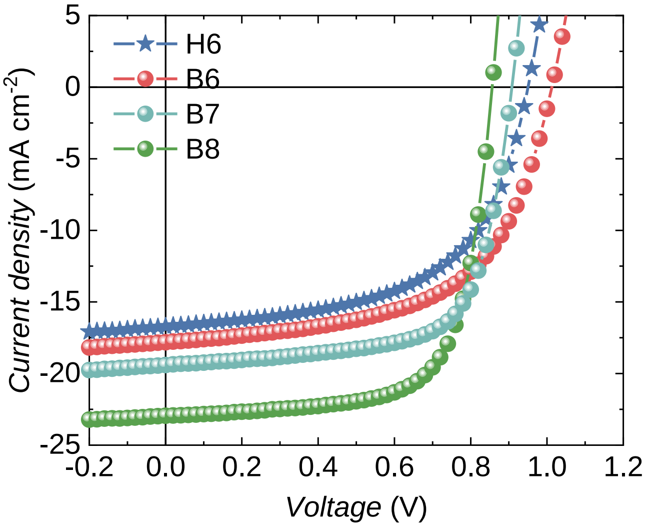


Supplementary Fig. 9. **J-V curves: ITIC based OSC.** Representative current density-Voltage (J-V) characteristics of devices comprising the hexanary blend H6, as well as B6-B8. Source data are provided as a Source Data file.

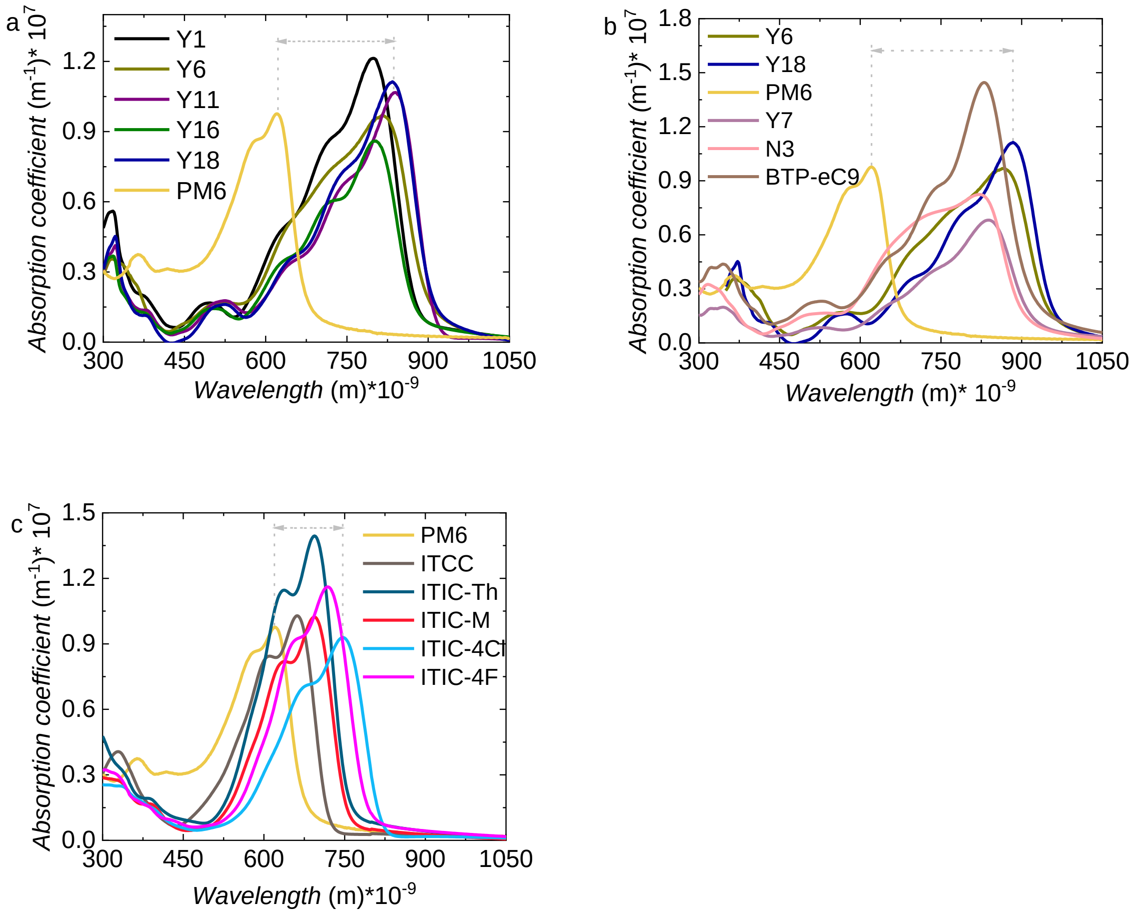


Supplementary Fig. 10. **Absorption coefficient** of donor and acceptors used in H1 **a**, H5 **b** and H6 **c**. Source data are provided as a Source Data file.

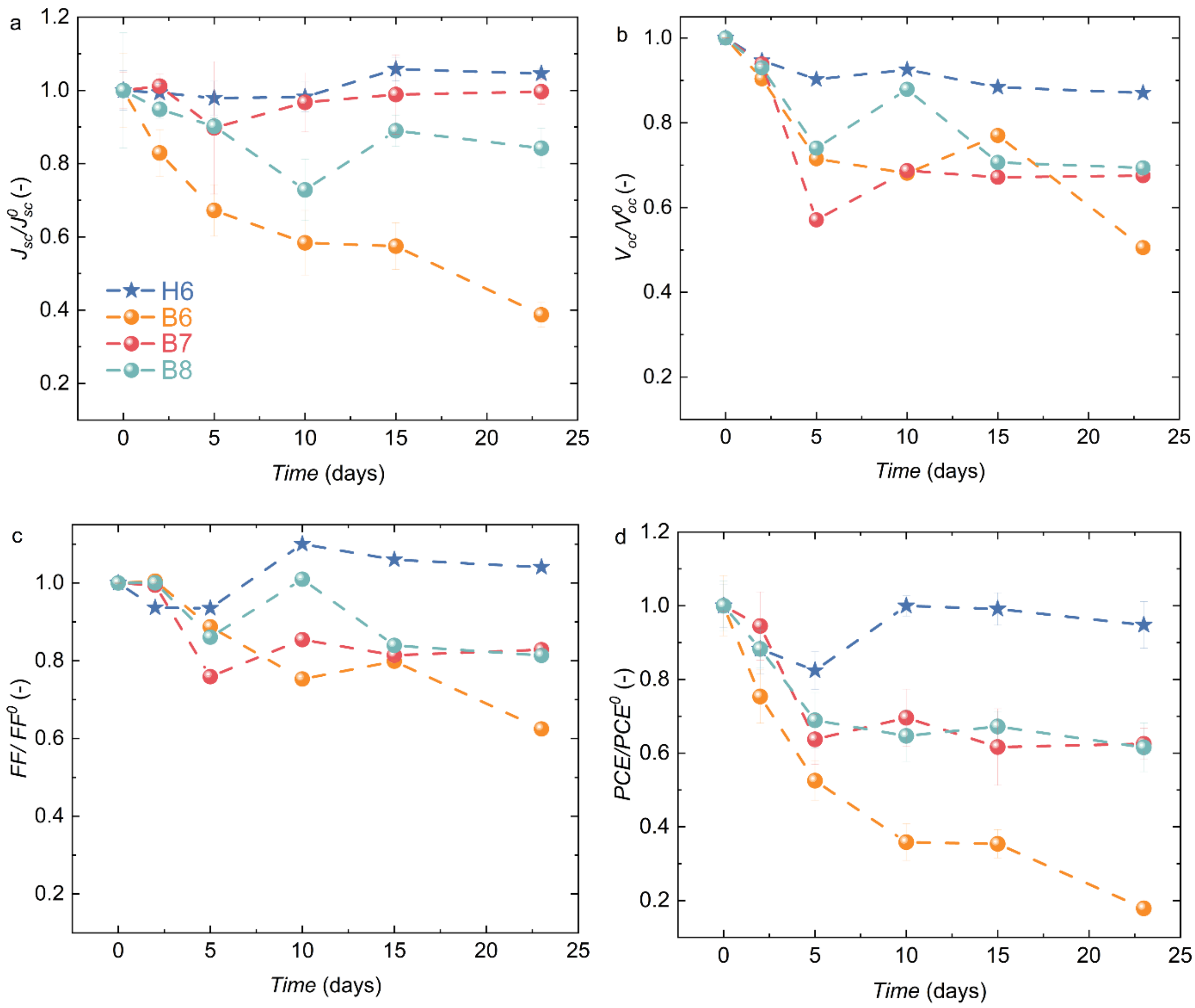


Supplementary Fig. 11. **Thermal aging studies of ITIC based OSC a** $J_{sc}$, **b** $V_{oc}$, **c** FF and **d** PCE of corresponding devices recorded after annealing up to 23 days at 130 °C; data points represent the mean and standard deviation of measurements done for up to 6 pixels on the same substrate. Source data are provided as a Source Data file.

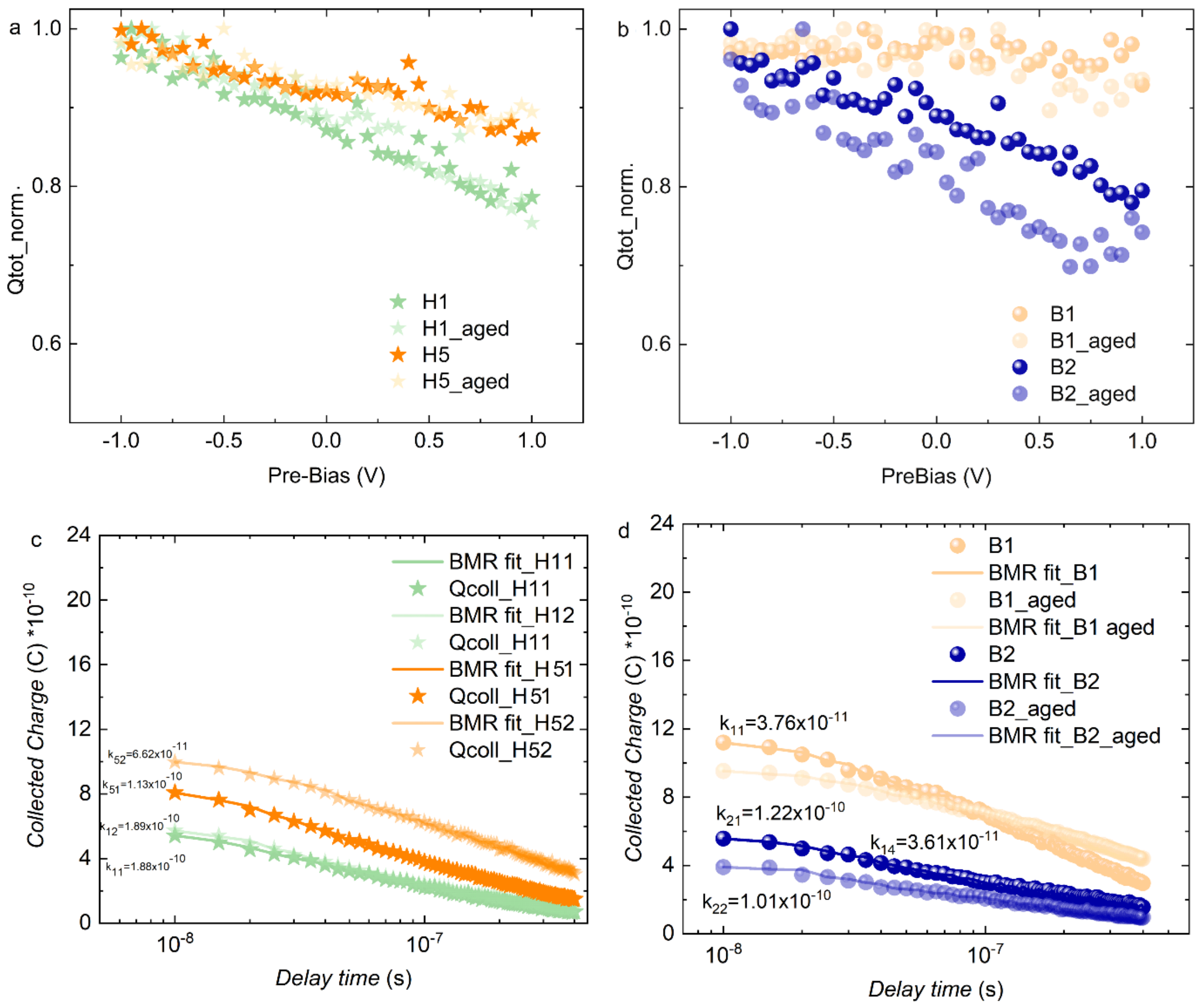


Supplementary Fig. 12. **Field dependence and decay of the charge generated in as cast and aged devices.** Total extracted charge ($Q_{tot}$) as a function of the applied pre-bias of H1 **a** and B1 **b** devices during laser excitation at 532 nm, normalized to $Q_{tot}$ at -1 V. The pulse fluence was 0.03 µJ/cm$^2$ and the time delay was 15 ns. Recombination dynamics at $V_{pre} = V_{oc}$ for different excitation fluences of the binary and hexanary devices with a 100 nm active layer thickness and an active area of 1 mm$^{2.}$ hexanary systems **c**; binary systems **d**. The bimolecular recombination coefficient is determined by fitting to the equation: $Qcoll(td + \Delta t) = Qcoll(td) - [Qpre(td + \Delta t) - Qpre(td)] - k\frac{Q^2coll(td)+2Qcoll(td)Qdark}{eAd}\Delta t$. Source data are provided as a Source Data file.

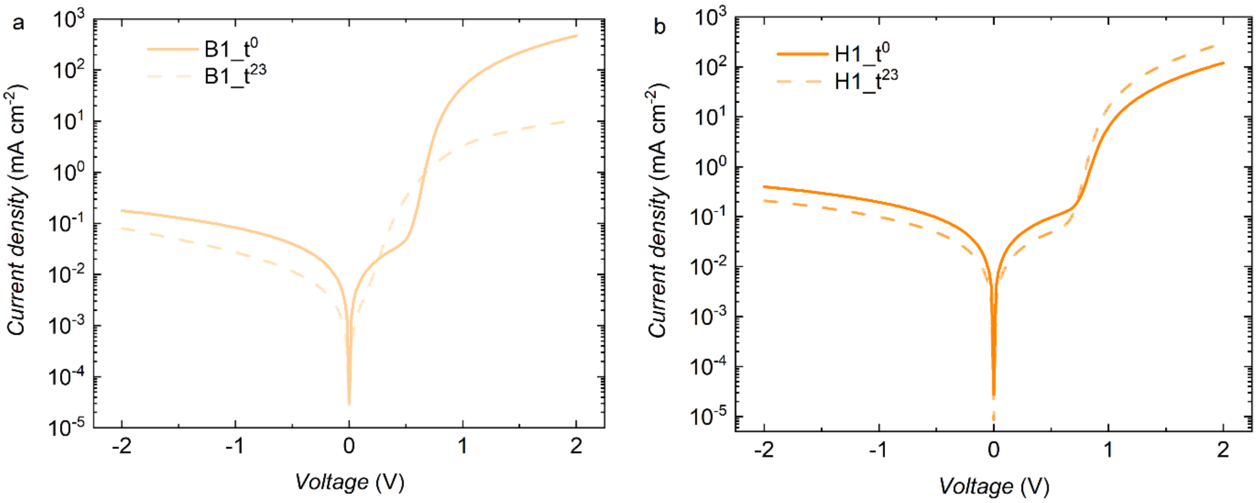


Supplementary Fig. 13. **JV curves of OSC in dark.** The dark JV curves of fresh and aged devices of **a** B1 and **b** H1 device. Source data are provided as a Source Data file.

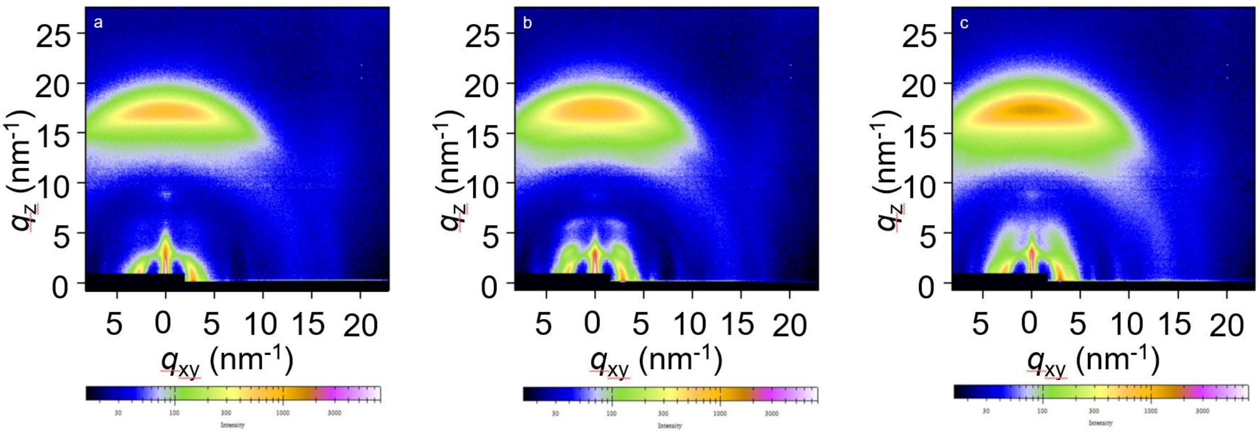


Supplementary Fig. 14. **GIWAXS patterns** of **a** PM6:Y6, **b** PM6:Y6:Y11:Y16 and **c** PM6:Y1:Y6:Y11: Y16:Y18 after spin-coated from chloroform and annealed at 120 $^{o}$C for 1 min, followed by ageing for 120 h at 130 $^{o}$C. The thickness of all the GIWAXS conducted films can be found in Supplementary Table 5.

Supplementary Table 6. **Thickness of the films** used for GIWAXS studies.

| Sample. No. | Film name | Additives/annealing | Thickness, nm |
|---|---|---|---|
| 01 | Si substrate/PM6 | No/ 120 ºC, 1 min. | 133 |
| 02 | Si substrate/Y6 | No/No | 50 |
| 03 | Si substrate/Y6 | No/ 120 ºC, 1 min. | 60 |
| 04 | Si substrate/Y6:Y11:Y16 | No/No | 113 |
| 05 | Si substrate/ Y6:Y11:Y16 | No/ 120 ºC, 1 min. | 113 |
| 06 | Si substrate/ Y1:Y6:Y11:Y16:Y18 | No/No | 107 |
| 07 | Si substrate/ Y1:Y6:Y11:Y16:Y18 | No/ 120 ºC, 1 min. | 107 |
| 08 | Si substrate/ Y1:Y6:Y18 | No/ No | 81 |
| 09 | Si substrate/ PM6:Y6:Y11:Y16 | No/ 120 ºC, 1 min. | 131 |
| 10 | Si substrate/ PM6:Y1:Y6:Y11:Y16:Y18 | No/ 120 ºC, 1 min. | 125 |
| 11 | Si substrate/ PM6:Y6 | No/120 ºC, 1 min. | 72 |
| 12 | Si substrate/ PM6:Y6:Y11:Y16 | 0,5% CN/ No | 141 |
| 13 | Si substrate/ PM6:Y1:Y6:Y11:Y16:Y18 | 0,5% CN/ No | 201 |
| 14 | Si substrate/ PM6:Y6 | 0,5% CN/ No | 124 |

Supplementary Table 7. **The thickness of the active layer films** referred to in figure 5 of the main text.

| Sample no. | Device name | Thickness, nm |
|---|---|---|
| 01 | H1_1 | 43 |
| 02 | H1_2 | 112 |
| 03 | H1_3 | 390 |
| 04 | B2_1 | 38 |
| 05 | B2_2 | 90 |
| 06 | B2_3 | 310 |

*Supplementary* Table 8. **Mean and standard deviation of photovoltaic device parameters** for 6 devices on the same substrate. H1 and B2 of fresh and aged devices with different active layer thickness. The donor:acceptor ratios are weight ratios.

| | | as prepared | | | | aged at 130 ºC for 336 h | | | |
|---|---|---|---|---|---|---|---|---|---|
| Film Thickness (nm) | | $J_{sc}$ (mA cm$^{-2}$) | $V_{oc}$ (V) | FF (%) | PCE (%) | $J_{sc}$ (mA cm$^{-2}$) | $V_{oc}$ (V) | FF (%) | PCE (%) |
| H1 | 43 | 13.8 ± 0.4 | 892 ± 1 | 62.2 ± 1 | 7.7 ± 0.3 | 15.8 ± 0.3 | 834 ± 8 | 60.5 ± 3 | 7.9 ± 0.3 |
| | 112 | 21.8 ± 0.3 | 895 ± 1 | 59.2 ± 1 | 11.6 ± 0.3 | 22.2 ± 0.5 | 859 ± 2 | 56.8 ± 2 | 10.8 ± 0.5 |
| | 390 | 21.3 ± 0.6 | 703 ± 6 | 51.3 ± 1 | 7.7 ± 0.2 | 22.9 ± 0.1 | 746 ± 0 | 42.5 ± 0 | 7.3 ± 0.0 |
| B2 | 40 | 15.2 ± 0.5 | 864 ± 2 | 68.8 ± 1 | 9.1 ± 0.3 | 15.5 ± 0.4 | 784 ± 20 | 61.1 ± 3 | 7.5 ± 0.3 |
| | 90 | 25.3 ± 0.4 | 872 ± 2 | 65.9 ± 1 | 14.5 ± 0.3 | 5.8 ± 0.4 | 748 ± 20 | 46.1 ± 3 | 2.0 ± 0.3 |
| | 310 | 20.2 ± 0.7 | 770 ± 3 | 53.8 ± 1 | 8.4 ± 0.3 | 20.5 ± 0.1 | 633 ± 1 | 49.8 ± 0 | 6.5 ± 0.0 |

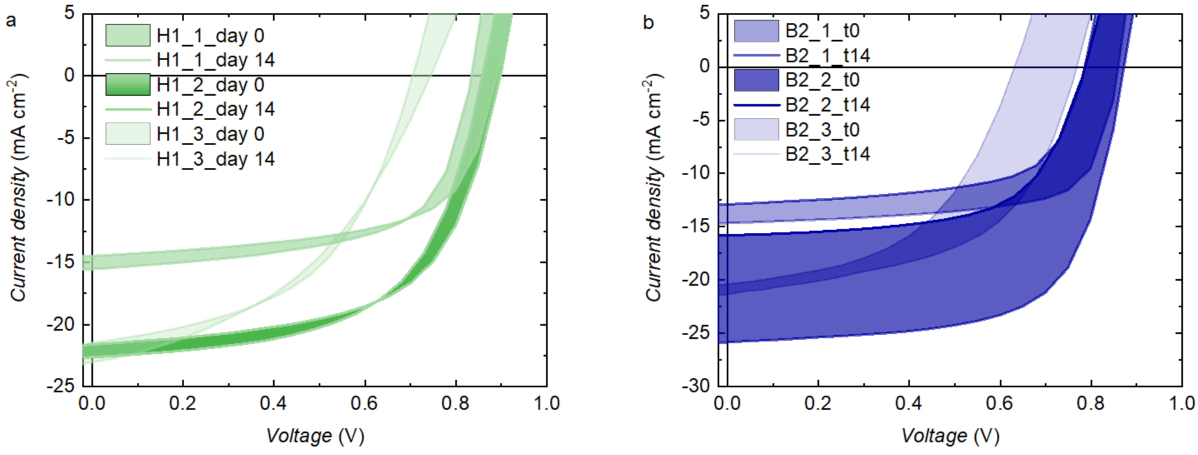


Supplementary Fig. 15. **J-V curves of devices with varied active layer thickness.** Representative J-V curves of H1 **a** and B2 **b** with different active layer thickness. Source data are provided as a Source Data file.

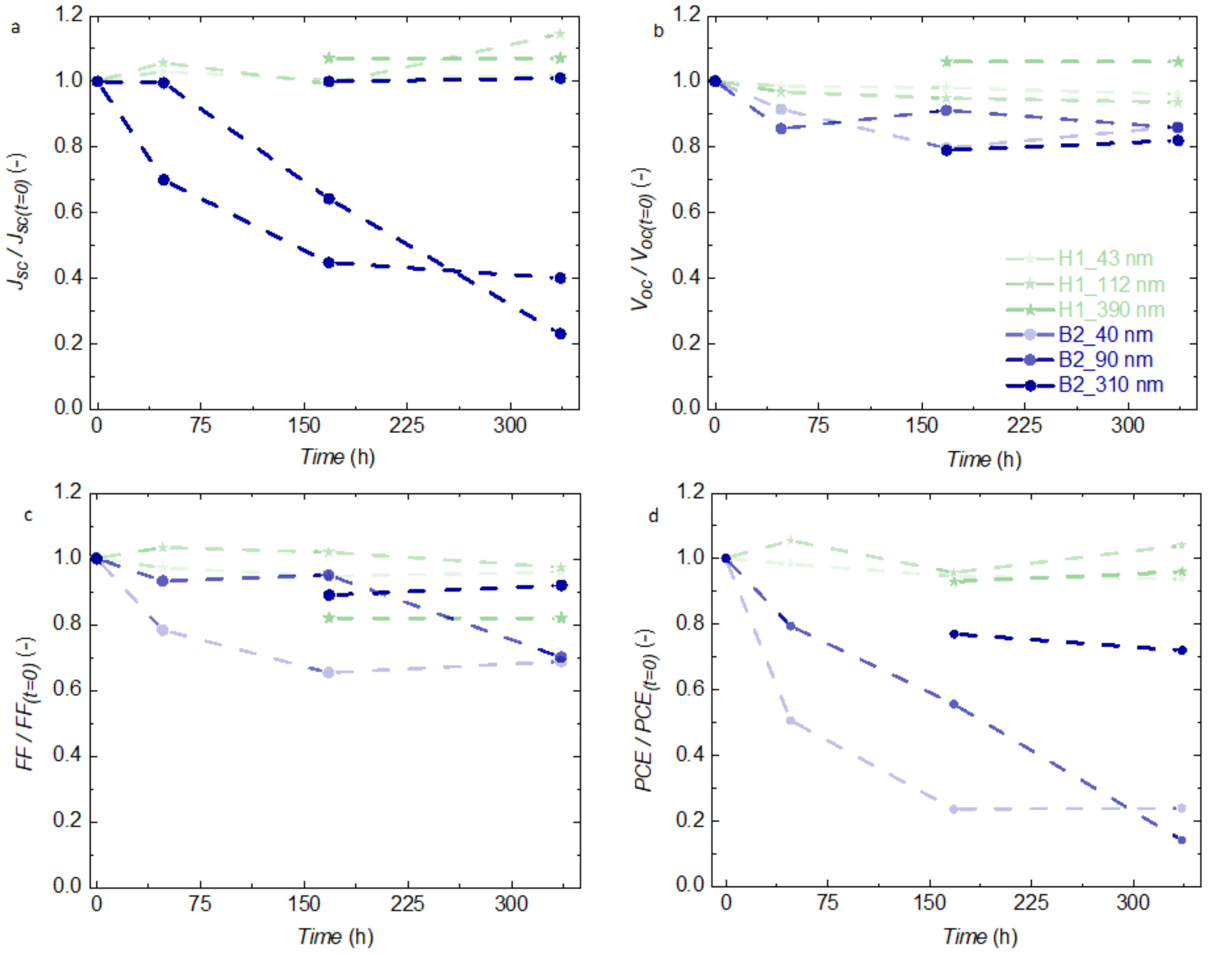


Supplementary Fig. 16. **Thermal aging studies with varied active layer thickness. a** $J_{sc}$, **b** $V_{oc}$, **c** FF and **d** PCE of H1, and B2 devices with different active layer thickness recorded at regular intervals by annealing half devices up to 336 h (14 days) at 130 °C; data points represent the mean and standard deviation of measurements done for up to 6 pixels on the same substrate. Source data are provided as a Source Data file.

## Supplementary References


1. Bertrandie, J. et al. The Energy Level Conundrum of Organic Semiconductors in Solar Cells. *Adv. Mater.* **34**, 2202575 (2022).
2. Hultmark, S. et al. Suppressing Co-Crystallization of Halogenated Non-Fullerene Acceptors for Thermally Stable Ternary Solar Cells. *Adv. Funct. Mater.* **30**, 2005462 (2020).
