# Supplementary material for: Hexanary blends: a strategy towards thermally stable organic photovoltaics
Source: Nat Commun. 2023 Aug 1;14:4608. doi: 10.1038/s41467-023-39830-6 (PMC10393981; doi:10.1038/s41467-023-39830-6)
Supplement: Supplementary file 3 — Reporting Summary [file 41467_2023_39830_MOESM3_ESM.pdf]

nature research

Corresponding author(s): Sri Harish Kumar Paleti, Christian Müller, Derya Baran

Solar Cells Reporting Summary

Nature Research wishes to improve the reproducibility of the work that we publish. This form is intended for publication with all accepted papers reporting the characterization of photovoltaic devices and provides structure for consistency and transparency in reporting. Some list items might not apply to an individual manuscript, but all fields must be completed for clarity.

For further information on Nature Research policies, including our data availability policy, see Authors & Referees.

Experimental design

Please check: are the following details reported in the manuscript?

1. Dimensions

Item	Yes	No	Response
Area of the tested solar cells	☐	☒	The area of each pixel is 0.1 cm^2.
Method used to determine the device area	☐	☒	The area of the pixel is determined from the layout of ITO substrate and top contact mask.

2. Current-voltage characterization

Item	Yes	No	Response
Current density-voltage (J-V) plots in both forward and backward direction	☐	☒	So far, organic solar cells literature did not report any hysteresis. Hence measurements were conducted only in forward direction.
Voltage scan conditions<br>*For instance: scan direction, speed, dwell times*	☐	☒	scan step is 20 mV and 10 ms. The dependency of the efficiency on the scan conditions is not reported in the Organic solar cells literature. Hence not mentioned in the manuscript.
Test environment<br>*For instance: characterization temperature, in air or in glove box*	☒	☐	In the experimental section
Protocol for preconditioning of the device before its characterization	☐	☒	No preconditioning of the devices was done prior to their characterization.
Stability of the J-V characteristic<br>*Verified with time evolution of the maximum power point or with the photocurrent at maximum power point; see ref. 7 for details.*	☒	☐	Stability data of PV parameters can be found in fig.4, fig.5, fig. S8, fig. S11, fig. S13, fig. S15 and fig. S16.

3. Hysteresis or any other unusual behaviour

Item	Yes	No	Response
Description of the unusual behaviour observed during the characterization	☐	☒	No unusual behavior observed
Related experimental data	☐	☒	No unusual behavior observed

4. Efficiency

Item	Yes	No	Response
External quantum efficiency (EQE) or incident photons to current efficiency (IPCE)	☐	☒	EQE's of the devices are not shown as the data is not relevant for the studies reported here. But, can be provided, if needed.
A comparison between the integrated response under the standard reference spectrum and the response measure under the simulator	☐	☒	EQE data is not shown in the manuscript.
For tandem solar cells, the bias illumination and bias voltage used for each subcell	☐	☒	Tandem devices are not fabricated in this study

5. Calibration

Item	Yes	No	Response
Light source and reference cell or sensor used for the characterization	☒	☐	AM1.5G spectra was simulated using LED's (WaveLab SINUS-70). Si reference cell from Newport with KG5 filter on it.
Confirmation that the reference cell was calibrated and certified	☒	☐	The calibration was conducted by Newport in September 2020 and valid for 5 years

Calculation of spectral mismatch between the reference cell and the devices under test

- [ ] Yes
- [x] No

The spectral mismatch was measured during EQE readings. But, data not included as it is not critical for the claims and conclusions of this study.

6. Mask/aperture

Size of the mask/aperture used during testing

- [x] Yes
- [ ] No

The measured mask area used in this study is 0.1 cm^2

Variation of the measured short-circuit current density with the mask/aperture area

- [ ] Yes
- [x] No

not relevant to this study, but the device area has been kept constant for all the pixels used for this study.

7. Performance certification

Identity of the independent certification laboratory that confirmed the photovoltaic performance

- [ ] Yes
- [x] No

Not applicable

A copy of any certificate(s)
Provide in Supplementary Information

- [ ] Yes
- [x] No

Not applicable

8. Statistics

Number of solar cells tested

- [x] Yes
- [ ] No

6 cells per system was tested. Details provided in the captions of table 1.

Statistical analysis of the device performance

- [x] Yes
- [ ] No

Values provided in the table 1 and table S4

9. Long-term stability analysis

Type of analysis, bias conditions and environmental conditions
For instance: illumination type, temperature, atmosphere humidity, encapsulation method, preconditioning temperature

- [x] Yes
- [ ] No

The environmental and thermal annealing conditions are mentioned in the experimental section and the results section.
